# Contribution of glutamatergic projections to neurons in the nonhuman primate substantia nigra pars reticulata for reactive inhibition

**DOI:** 10.1073/pnas.2427032122

**Published:** 2025-06-26

**Authors:** Atsushi Yoshida, Okihide Hikosaka

**Affiliations:** ^a^Neuronal Networks Section, Laboratory of Sensorimotor Research, National Eye Institute, National Institutes of Health, Bethesda, MD 20892; ^b^Systems Neuroscience Laboratory, Department of Physiology, Hokkaido University Graduate School of Medicine, Sapporo, Hokkaido 060-8638, Japan

**Keywords:** basal ganglia function, substantia nigra pars reticulata, glutamatergic inputs, action selection and suppression

## Abstract

The basal ganglia play a crucial role in action selection by facilitating desired movements and suppressing unwanted ones. The substantia nigra pars reticulata (SNr), a key output nucleus, facilitates movement through disinhibition of the superior colliculus (SC). However, its role in action suppression, particularly in primates, remains less clear. We investigated whether individual SNr neurons in three male macaque monkeys bidirectionally modulate their activity to both facilitate and suppress actions and examined the role of glutamatergic inputs in suppression. Monkeys performed a sequential choice task, selecting or rejecting visually presented targets. Electrophysiological recordings showed that SNr neurons decreased firing rates during target selection and increased firing rates during rejection, demonstrating bidirectional modulation. Pharmacological blockade of glutamatergic inputs to the lateral SNr disrupted saccadic control and impaired suppression of reflexive saccades, providing causal evidence for the role of excitatory input in behavioral inhibition. These findings suggest that glutamatergic projections, potentially originating from sources including the subthalamic nucleus, contribute to the increased SNr activity during action suppression. Our results highlight conserved basal ganglia mechanisms across species and offer insights into the neural substrates of action selection and suppression in primates, with implications for understanding disorders such as Parkinson’s disease.

## Significance

Understanding how the basal ganglia facilitate desired actions while suppressing unwanted ones is fundamental to neuroscience. This study shows that neurons in the primate substantia nigra pars reticulata (SNr) bidirectionally modulate activity to control action, decreasing firing rates to facilitate movements and increasing rates to suppress them. Importantly, we provide causal evidence that glutamatergic inputs to the lateral SNr mediate action suppression. These findings reveal a conserved mechanism of action control in primates and highlight the role of excitatory inputs in behavioral inhibition. This advances our understanding of basal ganglia function and has significant implications for treating movement disorders like Parkinson’s disease.

Our ability to select desired actions while suppressing unwanted ones is essential. The basal ganglia play a critical role in this selective control, with the substantia nigra pars reticulata (SNr) as a major output nucleus (1–6). The SNr tonically inhibits the superior colliculus (SC), a key structure for saccadic eye movements, through GABAergic projections (7–19). During saccades, SNr neurons decrease firing, disinhibiting SC neurons and enabling movement (20–32). While the SNr is known to facilitate desired movements through the disinhibition of downstream targets, its role in suppressing unwanted actions, particularly in primates, remains unclear. Rodent studies suggest increased lateral SNr activity during movement cancellation, possibly driven by excitatory inputs from the subthalamic nucleus (STN) (33–38). However, to our knowledge, no study to date has investigated whether individual SNr neurons in primates exhibit a bidirectional coding scheme that supports both facilitation and suppression of actions.

If the SNr is involved in behavioral inhibition, another critical question arises: Does it mediate proactive inhibition, reactive inhibition, or both? Reactive inhibition is typically a rapid, stimulus-driven response, an immediate suppression of a prepotent, reflexive saccade triggered by sudden sensory input. In contrast, proactive inhibition is an anticipatory, goal-directed control mechanism that prepares the system to suppress actions that conflict with current behavioral objectives, such as when an individual intentionally ignores distracting stimuli during a focused task (39). The basal ganglia have been implicated in both forms of inhibition, with theories suggesting a division of labor: The indirect pathway may primarily support proactive inhibition, while the hyperdirect pathway may be more involved in reactive inhibition (39). Our previous work has provided evidence for the involvement of the anterior striatum, a key component of the indirect pathway in proactive control (40). Given that the SNr is a major output nucleus of the basal ganglia, receiving inputs from both the indirect and hyperdirect pathways, it is crucial to determine whether its bidirectional coding reflects proactive inhibition, reactive inhibition, or a combination of both.

In this study, we test the hypothesis that individual SNr neurons in primates bidirectionally modulate activity to facilitate and suppress actions, decreasing during target selection and increasing during rejection. We further investigate whether the increased activity observed during rejection reflects reactive inhibition or proactive inhibition. Moreover, we hypothesize that the increased activity during rejection involves excitatory, glutamatergic input to the SNr, with the STN representing a principal candidate source for this input.

To evaluate these hypotheses, we recorded SNr neuronal activity in macaque monkeys performing a sequential choice task, where the monkeys either selected or rejected visually presented targets, as well as in a fixation task designed to require the suppression of reflexive saccades (40). Using electrophysiology and pharmacological blockade of glutamatergic inputs to the lateral SNr, we observed that SNr neurons exhibited bidirectional modulation during task performance, with decreased activity during target selection and increased activity during rejection. Using electrophysiology and pharmacological manipulation targeting glutamatergic inputs to the lateral SNr, we examined the relationship between SNr activity, behavioral choice, and the causal role of these inputs in saccade control and suppression. Our findings, detailed below, reveal bidirectional modulation linked to action selection or rejection and provide evidence for the crucial contribution of glutamatergic transmission to reactive inhibition.

## Results

### Behavior Results in the Choice of Task.

**Fig. 1.**
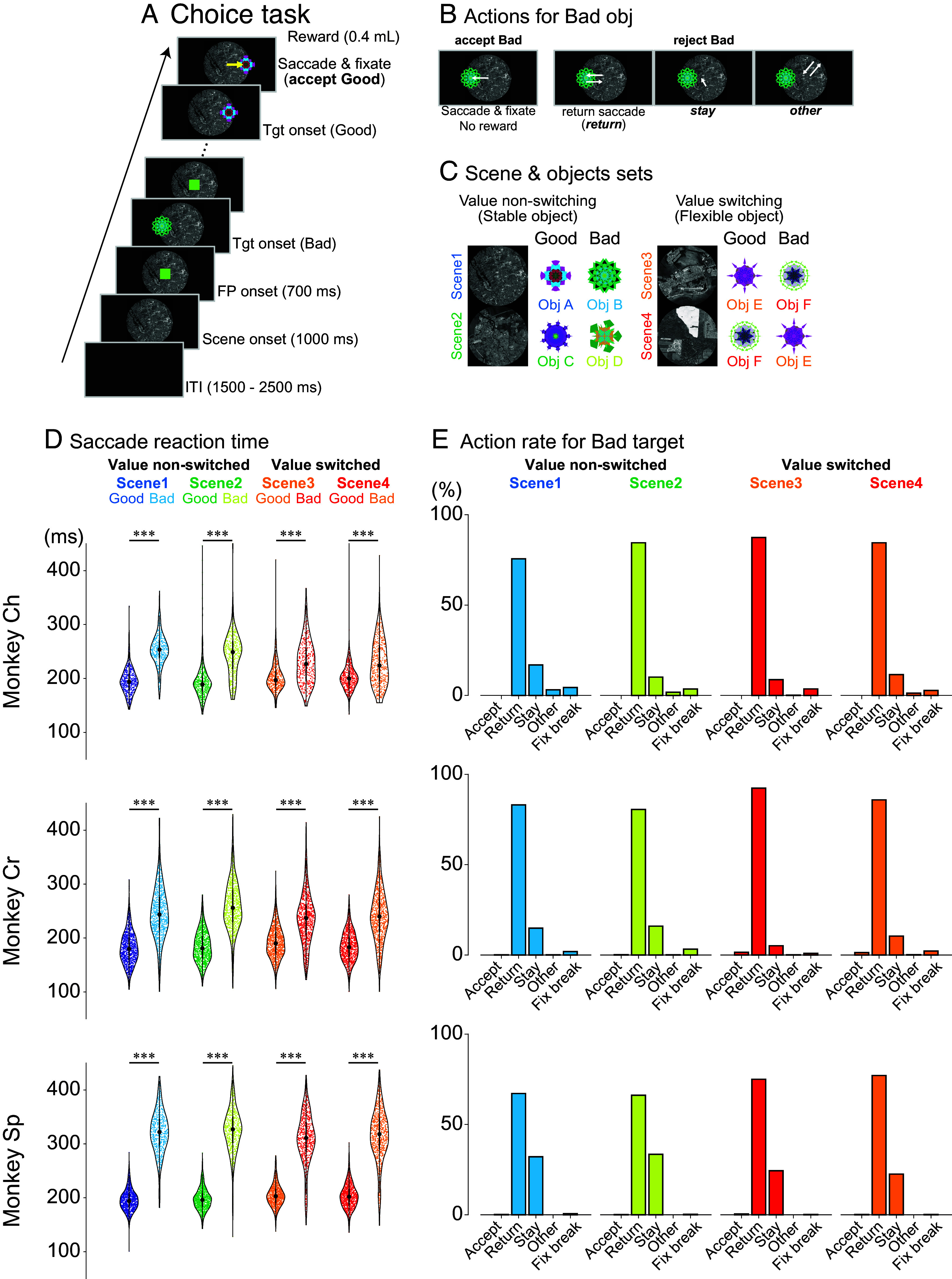
Choice task paradigm and behavioral performance. (*A*) Time course of the choice task. A background scene is initially presented (1,000 ms), followed by a fixation point (FP, 700 ms). Subsequently, either a good (rewarded) or bad (nonrewarded) object is randomly presented at one of six possible locations (0°, 45°, 135°, 180°, 225°, or 315°). (*B*) Possible behavioral responses to bad objects. When a bad object appears, monkeys can either incorrectly accept it by making a saccade and fixating on it (accept bad, resulting in no reward) or correctly reject it through one of three actions: making a saccade toward the object and immediately returning to center (return), maintaining fixation near the center without making a saccade (stay) or making a saccade in a direction different from the object’s location (other). (*C*) Scene and object combinations used for neuronal recordings. Each recording session utilized one of six sets, with each set containing four scenes. Each scene was associated with two objects: one good (rewarded) and one bad (nonrewarded). In scenes 1 and 2, object values remained constant (value nonswitching), while in scenes 3 and 4, the same objects were used but with reversed reward values (value-switching), illustrating context-dependent value assignment. (*D*) Saccadic reaction times for three monkeys (Ch, Cr, and Sp). Violin plots show that, across all scenes and monkeys, saccades to good objects had significantly shorter latencies compared to bad objects (***P < 0.0001, Welch’s *t* test). (*E*) Proportions of different behavioral responses to bad objects across all scenes. All monkeys exhibited similar patterns: Incorrect acceptance of bad objects was rare, with rejection responses predominantly consisting of return saccades, followed by stay responses and occasional other responses. Abbreviations: FP, fixation point; ITI, intertrial interval; Obj, object; Tgt, target.

Three monkeys were trained to perform a sequential choice task, evaluating and responding to objects based on learned values (Fig. 1*A*). Monkeys could accept an object by making a saccade and maintaining fixation or reject it through three alternatives: initiating a saccade before returning to the center (“return”), maintaining central gaze without a saccade (“stay”), or making a saccade away (“other”) (Fig. 1*B*). Upon rejection, the object disappeared, and a new one was presented until the object was accepted.

Each recording session began with one of four randomly selected scenes providing context on the value of two objects: a “good” object yielding a reward and a “bad” object yielding none (Fig. 1*C*). To ensure that neural responses reflected object value and not simply visual features, we implemented a value-switching design in scenes 3 and 4. This involved presenting the same pair of objects (Obj E and Obj F) in both scenes, but reversing their reward contingencies (Obj E good/Obj F bad in scene 3; Obj E bad/Obj F good in scene 4). This allowed us to isolate the effect of value on neural activity while controlling for visual input.

Neural recordings commenced after monkeys achieved stable performance, reliably distinguishing good from bad objects with 90% accuracy across scenes (*SI Appendix*, Fig. S1). To avoid bias, one of six object-scene sets was randomly selected for each session (*SI Appendix*, Fig. S1).

Saccadic reaction time analysis revealed faster responses to good objects compared to bad ones across all four scenes (Welch’s *t* test, *P* < 0.0001; *SI Appendix*, Table S1). This consistent difference indicates that monkeys recognized object values before initiating saccadic responses (Fig. 1*D*).

When rejecting bad objects, monkeys predominantly used the return saccade strategy (Fig. 1*E*), making a saccade toward the bad object and quickly returning their gaze to the center. This accounted for most rejections across all scenes (monkey Ch: 75.6 to 87.4%; monkey Cr: 80.5 to 92.3%; monkey Sp: 66.2 to 77.1%; see *SI Appendix*, Table S2 for details). The stay strategy, where monkeys maintained central fixation without making a saccade, was less frequent (monkey Ch: 8.7 to 16.9%; monkey Cr: 5.1 to 16.0%; monkey Sp: 22.5 to 33.5%). It is important to note that, while both “return” and “stay” strategies lead to the rejection of a bad object, they differ fundamentally in their motor output: The “return” strategy involves a saccade, whereas the “stay” strategy does not. The “return” saccade, however, is not a reflexive response to the object; it is a deliberate action executed after the initial evaluation of the object’s value, reflecting the monkey’s decision to reject it. The preference for the return saccade likely reflects task timing: The stay response required maintaining fixation for 400 ms before the next target appeared, whereas the return response allowed the next target to appear immediately after returning gaze to the center, bypassing the fixed delay. This efficiency likely motivated monkeys to favor the return strategy. Comparing these two rejection strategies is crucial for understanding the role of the SNr, as it enables us to dissociate neural activity associated with saccade execution from that linked to the cognitive process of rejecting an undesirable object.

The proportion of stay responses differed between scenes with stable (scenes 1 and 2) versus flexible, context-dependent values (scenes 3 and 4). Monkeys showed significantly more stay responses in scenes 1 and 2 compared to scenes 3 and 4 (Fisher’s exact test: monkey Ch, *P* < 9.60 × 10^−3^, φ = 0.06, 95% CI: 0.53 to 0.92; monkey Cr, *P* < 9.68 × 10^−14^, φ = 0.12, 95% CI: 0.38 to 0.58; monkey Sp, *P* < 5.22 × 10^−10^, φ = 0.11, 95% CI: 0.54 to 0.73). This difference may reflect the higher cognitive demand of processing context-dependent values in scenes 3 and 4.

### Neuronal Activity of SNr Neurons During the Choice Task.

The SNr is traditionally recognized for its role in controlling saccadic eye movements by inhibiting the SC. However, emerging evidence indicates that the SNr may also contribute to higher-order cognitive functions, such as action selection and behavioral inhibition. To investigate how SNr neurons encode object evaluation and action selection, we recorded single-unit activity from 129 neurons across three monkeys (29 from monkey Ch, 51 from monkey Cr, and 49 from monkey Sp) during the choice task. Neurons were included based on observed activity changes following target onset, specifically those with significant firing rate modulation.

**Fig. 2.**
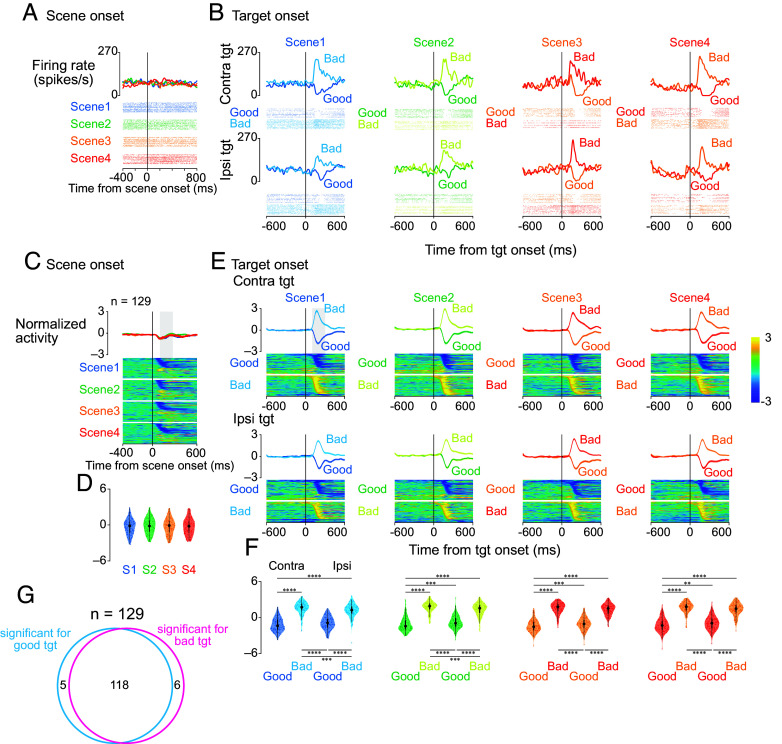
Single-neuron and population responses to scene and target presentation during the choice task. (*A* and *B*) Activity of a representative SNr neuron. Spike density functions (*Top*) and raster plots (*Bottom*) are shown aligned to scene onset (*A*) and target onset (*B*). In (*A*), different colors represent different scenes. In (*B*), activity is displayed separately for contralateral (*Upper*) and ipsilateral (*Lower*) target presentations, with different colors indicating responses to good and bad objects in each scene. (*C*) Population activity aligned to scene onset (n = 129 neurons). The upper panels show mean firing rates (±SEM, shaded area) for each scene. Lower panels display color-coded normalized firing rates of individual neurons (rows) over time. (*D*) Distribution of neuronal responses to scene onset. Violin plots quantify activity during a 200-ms window starting 100 ms after scene onset [gray rectangle in (*C*)]. Plot elements represent the median (large circle), interquartile range (thick line), and range (thin line). (*E*) Population activity aligned to target onset, shown separately for contralateral and ipsilateral presentations of good and bad objects across all scenes. The format is consistent with (*C*). (*F*) Distribution of neuronal responses to target presentation. Violin plots quantify activity during a 200-ms window starting 100 ms after target onset. Asterisks denote significant differences between conditions (*P < 0.05, **P < 0.01, ***P < 0.001, and ****P < 0.0001, post hoc pairwise *t* test with Bonferroni correction). (*G*) Venn diagram depicting the distribution of neurons with significant response modulation to target presentation in scene 1 (contralateral targets). Neural activity was compared between a 200-ms pretarget baseline period and a 200-ms window starting 100 ms after target onset. Of 129 neurons, 118 showed significant modulation to both good and bad objects, while 5 and 6 neurons responded exclusively to good or bad objects, respectively. Abbreviation: SEM, standard error of the mean.

A representative SNr neuron showed distinct activity patterns in response to good and bad objects (Fig. 2 *A* and *B*). While minimally responsive to scene onset (Fig. 2*A*), this neuron exhibited robust value-dependent modulation after target presentation: Firing rates decreased for good objects and increased for bad objects across all scenes (Fig. 2*B*). This bidirectional response pattern was consistent for both contralateral and ipsilateral target presentations.

Population analyses confirmed this response pattern as characteristic of SNr neurons (Fig. 2 *C* and *E*). Fig. 2 *C* and *E* illustrate the overall population activity, aligned by scene onset and target onset, respectively. Fig. 2*C* demonstrates that SNr neurons are minimally modulated by scene onset. Importantly, Fig. 2*E* reveals that SNr neurons exhibit bidirectional modulation following target onset, showing decreased activity for good objects and increased activity for bad objects. It is essential to note that the data in Fig. 2*E* encompass all bad object trials, irrespective of whether the monkey subsequently made a saccade (“return”) or withheld a saccade (“stay”). This methodological approach enables us to capture the overall response of SNr neurons to the presentation of a bad object, integrating both the visual response and the initial evaluative process. Normalized population activity showed minimal modulation at scene onset (Fig. 2*C*) but exhibited strong value-dependent responses following target presentation (Fig. 2*E*): decreased activity for good objects and increased activity for bad objects. Analysis within a 200-ms window (100-300 ms after target onset) revealed significant differences between good and bad object responses (parametric bootstrap tests for linear mixed-effects models with post hoc pairwise *t* test, Bonferroni-corrected; see *SI Appendix*, Table S3). Neural responses did not differ significantly across scenes (parametric bootstrap tests, full vs. null models; *P* = 0.44), suggesting activity reflected object values rather than visual features. This conclusion is supported by scenes 3 and 4, where identical objects elicited opposite responses based on context-dependent values.

Most SNr neurons exhibited bidirectional value coding. In scene 1, 91.5% (118/129) of neurons showed significant modulation to both good and bad objects during a 200-ms window (100-300 ms after target onset) compared to a 200-ms pretarget baseline (two-sample *t* test), while only 3.9% (5/129) and 4.7% (6/129) responded exclusively to good or bad objects, respectively (Fig. 2*G*). This suggests that individual SNr neurons dynamically encode both facilitation and suppression of actions through bidirectional modulation.

To assess whether SNr activity after target onset was related to saccade initiation, we realigned responses to saccade onset (*SI Appendix*, Fig. S2). Importantly, *SI Appendix*, Fig. S2 specifically focuses on “return” trials, where a saccade was executed. This targeted analysis enables us to examine the temporal relationship between SNr activity and saccade onset with greater precision. The neuron in Fig. 2*A* exhibited distinct temporal patterns for good versus bad objects (*SI Appendix*, Fig. S2*A*). For good objects, firing rates reached a minimum near saccade initiation, while for bad objects, peak activity occurred before saccade onset.

This pattern was consistent at the population level (*SI Appendix*, Fig. S2*B*). When accepting good objects, population activity showed maximal suppression around saccade initiation. In contrast, for bad objects (return saccades), peak activation occurred before movement onset. Statistical analysis confirmed significant differences between these perisaccadic response patterns (parametric bootstrap tests for linear mixed-effects models with Bonferroni-corrected post hoc *t* test; see *SI Appendix*, Table S4). Together, Fig. 2 and *SI Appendix*, Fig. S2 complement one another by providing both a broad overview of SNr bidirectional modulation and a detailed account of its temporal dynamics relative to saccade onset. This comprehensive approach reinforces our interpretation that SNr neurons modulate their activity to regulate action. These findings suggest that the increased SNr activity observed during bad object trials is not merely a consequence of saccade execution but instead reflects an anticipatory inhibitory process.

To examine the link between SNr activity and saccadic reaction times, we analyzed correlations between normalized firing rates (150 ms before to 50 ms after saccade onset) and normalized reaction times (*SI Appendix*, Fig. S2*D*). Reaction times were normalized due to skewed distributions for Bayesian linear mixed-effects models. A significant positive correlation was found only for contralateral bad objects [posterior mean β = 0.075, 95% credible interval (0.01, 0.14), P(β > 0) = 99.2%], while no significant correlations were observed for other conditions (contralateral good, ipsilateral good, ipsilateral bad; see *SI Appendix*, Table S5). The correlation between increased SNr activity and longer reaction times for contralateral bad objects aligns with the inhibitory role of SNr projections to the SC: Stronger SNr activation likely enhances SC suppression, delaying saccade initiation for contralateral targets. This further supports the notion that the increased SNr activity is associated with the suppression of unwanted movements.

### Neuronal Activity in the SNr During Choice Rejection and Saccade Suppression.

**Fig. 3.**
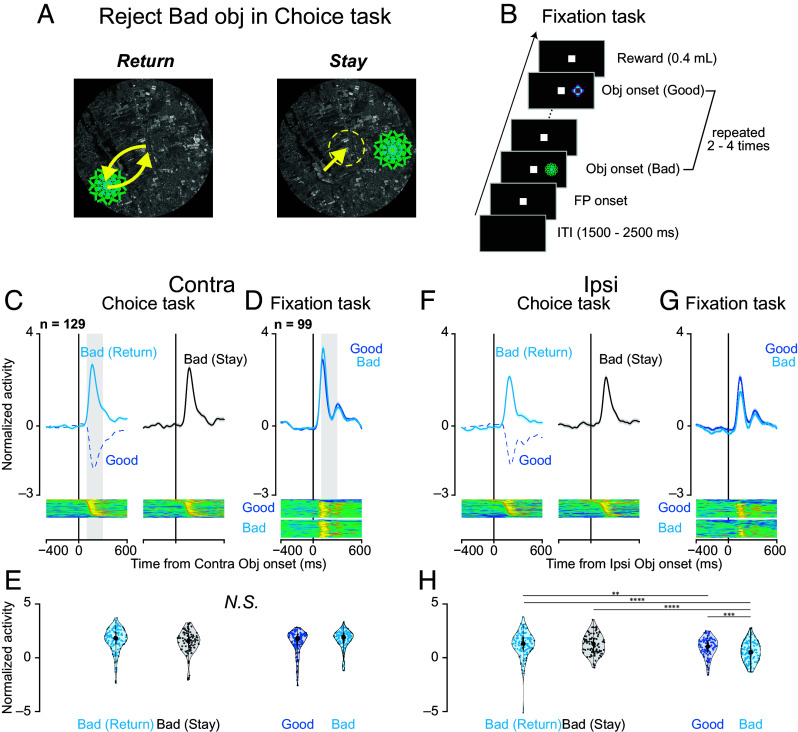
Neural activity during different rejection strategies and saccade suppression. (*A*) Schematic illustration of two rejection strategies for bad objects: “return” (saccade toward the object followed by return to center) and “stay” (maintaining central fixation). (*B*) Fixation task design. Objects from scene 1 of the choice task (both good and bad) were presented sequentially (2 to 4 presentations) in the contralateral or ipsilateral visual field. Monkeys were rewarded for maintaining central fixation throughout the presentations, requiring active suppression of reflexive saccades to the objects. (*C*) Population responses during bad object rejection in scene 1 for contralateral target presentations. Cyan traces represent “return” responses, black traces represent “stay” responses, and blue dotted traces represent responses during good object acceptance for comparison. Lower panels display color-coded normalized firing rates of individual neurons. (*D*) Population responses during the fixation task for contralateral object presentations. Blue traces represent responses to good objects, and cyan traces represent responses to bad objects. Lower panels show normalized single-neuron responses as in (*C*). (*E*) Distribution of neuronal responses during the choice task. Violin plots quantify activity during a 200-ms window starting 100 ms after object onset (gray rectangles in *C* and *D*). No significant differences were observed for contralateral presentations. (*F*) Population responses during bad object rejection in scene 1 for ipsilateral target presentations, displayed as in (*C*). (*G*) Population responses during the fixation task for ipsilateral object presentations, displayed as in (*D*). (*H*) Distribution of neuronal responses during the fixation task, quantified as in (*E*). For ipsilateral presentations, asterisks indicate significant differences between conditions (*P* < 0.05, *P < 0.01, **P < 0.001, and ***P < 0.0001, post hoc pairwise t test with Bonferroni correction).

To investigate the role of SNr in behavioral inhibition, we compared neuronal responses across rejection strategies and behavioral contexts. Monkeys rejected bad objects by either making a saccade toward the object and returning to the center (“return”) or maintaining central fixation without a saccade (“stay”) (Fig. 3*A*). While both strategies resulted in rejection, “return” involved saccade execution, and “stay” required saccade suppression. Fig. 3 directly compares SNr activity during “return” and “stay” trials. Notably, unlike Fig. 2*E*, which includes all bad object trials, Fig. 3 separates the data based on whether the monkey selected the “return” or “stay” strategy. This distinction enables us to determine whether the increased SNr activity observed during bad object presentation is associated with the execution of a specific motor response (i.e., saccade) or reflects a broader process of behavioral inhibition, such as rejecting the undesirable object.

Population activity during bad object rejection showed similar patterns between “return” and “stay” responses for both contralateral (Fig. 3*C*) and ipsilateral (Fig. 3*F*) presentations. Quantitative analysis of neuronal responses (200-ms window, 100 to 300 ms after object onset) revealed no significant differences between strategies (parametric bootstrap tests for linear mixed-effects models with Bonferroni -corrected post hoc *t* test; Fig. 3 *E* and *H*). This pivotal finding suggests that the increased SNr activity observed during bad object trials is not merely related to the execution or suppression of a specific saccade but rather reflects a broader process of behavioral inhibition, specifically the rejection of an undesirable object.

To examine SNr involvement in saccade suppression, we recorded 99 of the 129 neurons during a fixation task (Fig. 3*B*). Monkeys suppressed reflexive saccades to sequentially presented objects (2 to 4 presentations) while maintaining central fixation to earn a reward. The same objects from scene 1 of the choice task were used in the fixation task to control for visual features and isolate the effect of behavioral context.

During the fixation task, SNr neurons exhibited increased activity following object presentation in both contralateral (Fig. 3*D*) and ipsilateral (Fig. 3*G*) directions. Notably, this increase occurred even for objects labeled as “good” in the choice task, which had previously reduced SNr activity and facilitated saccadic responses (dotted lines in Fig. 3 *C* and *F*). The activity increase during central fixation in the fixation task was comparable to that observed during rejection in the choice task, with no significant differences for contralateral presentations (Fig. 3*E*) and some significant differences for ipsilateral comparisons (Fig. 3*H*; see *SI Appendix*, Table S6 for details).

Comparing the fixation and choice tasks highlights their distinct behavioral demands. The fixation task primarily challenges the monkey to reactively inhibit reflexive saccades to suddenly presented objects without evaluating their value. In contrast, the choice task involves a more complex process: Reactive inhibition allows time to assess object value, followed by proactive inhibition (rejecting undesirable objects) or rapid disinhibition (accepting desirable objects). This comparison underscores that the fixation task isolates basic reactive suppression, while the choice task builds on this mechanism to incorporate value-based decision-making and guide action selection.

### Effects of Glutamatergic Receptor Antagonists on the SNr.

Our neurophysiological findings suggest that increased SNr activity during both proactive inhibition (rejecting bad objects in the choice task) and reactive inhibition (suppressing reflexive saccades in the fixation task) may be driven by excitatory inputs to the SNr. To test this hypothesis and establish a causal link between glutamatergic transmission in the SNr and behavioral inhibition, we conducted local pharmacological manipulation of glutamatergic transmission in the lateral SNr, where most task-related neurons are located. This approach complements previous studies, such as Hikosaka and Wurtz (1984), which investigated the role of SNr output (GABAergic projections) in saccade control (41). In contrast, our experiment directly targets the input to the SNr, providing insights into the mechanisms underlying behavioral inhibition.

**Fig. 4.**
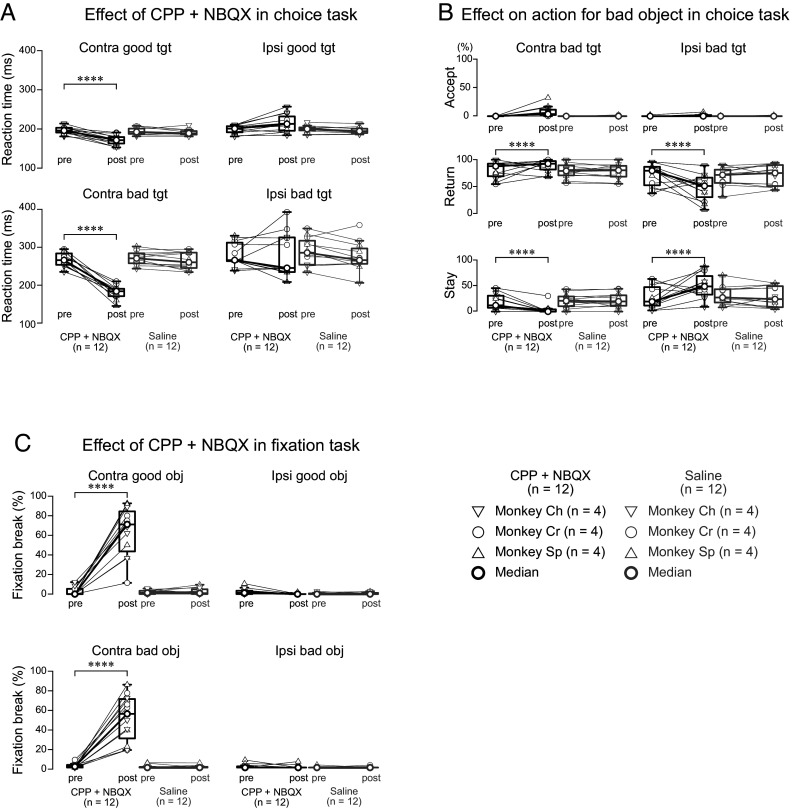
Behavioral effects of glutamatergic receptor antagonists during choice and fixation tasks. (*A*) Saccadic reaction times before (pre) and after (post) local injection of CPP/NBQX mixture or saline. Data points represent median values for individual sessions from three monkeys (inverted triangles: monkey Ch; circles: monkey Cr; triangles: monkey Sh; n = 4 sessions per condition per monkey). Thick circles and lines indicate population medians. Following CPP/NBQX injection, reaction times significantly decreased for both good and bad objects presented contralaterally. (*B*) Proportions of different actions (accept, return, stay) toward bad objects before and after drug injection. Format as in (*A*). CPP/NBQX injection significantly increased the proportion of return responses while decreasing stay responses for contralateral targets, with the opposite pattern observed for ipsilateral targets. (*C*) Fixation break error rates during the fixation task before and after injection. Format as in (*A*). CPP/NBQX significantly increased fixation break errors for both good and bad objects presented contralaterally. Asterisks denote significant differences (*P < 0.05, **P < 0.01, ***P < 0.001, and ****P < 0.0001, post hoc pairwise *t* test with Bonferroni correction). Abbreviations: CPP, (±)-3-(2-carboxypiperazin-4-yl) propyl-1-phosphonic acid (NMDA receptor antagonist); NBQX, 2,3-dihydroxy-6-nitro-7-sulfamoyl-benzo[f]quinoxaline (AMPA receptor antagonist); Tgt, target.

We injected glutamate receptor antagonists (a mixture of N-methyl-d-aspartate (NMDA) receptor antagonist (carboxypiperazin-4-propyl-1-phosphonic acid, CPP) and aminomethylphosphonic acid (AMPA) receptor antagonist (2,3-dihydroxy-6-nitro-7-sulfamoyl-benzo (F)quinoxaline, NBQX)) into the lateral SNr while monkeys performed both the choice and fixation tasks. The effects of this manipulation were striking and direction-specific. In the choice task, reaction times for contralateral saccades decreased significantly for both good and bad objects (Fig. 4*A*; *P* < 0.0001 for both comparisons, parametric bootstrap tests for generalized linear mixed-effects models with Bonferroni correction, see *SI Appendix*, Table S7). More critically, the manipulation altered rejection patterns: For contralateral bad objects, monkeys showed more “return” responses and fewer “stay” responses, while the opposite pattern occurred for ipsilateral bad objects (Fig. 4*B*; *P* < 0.0001 for all comparisons, see *SI Appendix*, Table S8).

The most prominent effect occurred in the fixation task, where glutamate receptor blockade severely impaired reflexive saccade suppression. After antagonist injection, monkeys showed a significant increase in fixation break errors for contralateral object presentations (Fig. 4*C*; *P* < 0.0001, see *SI Appendix*, Table S9). These effects were specific to glutamate receptor blockade, as saline injections caused no significant behavioral changes in either task (*SI Appendix*, Tables S7–S9).

These findings suggest that glutamatergic transmission in the lateral SNr plays a pivotal role in reactive inhibition. The shortened reaction times for both good and bad objects in the choice task, as well as the increased fixation break errors in the fixation task, indicate that blocking glutamatergic input disrupts the initial suppression of reflexive saccades. The lateralized effects, particularly the alterations in contralateral saccade behavior, align with the well-established role of the SNr in controlling contralateral eye movements. A more in-depth exploration of the potential mechanisms underlying these effects, including a proposed two-stage inhibition process and the involvement of specific pathways, will be further discussed in the *Discussion*.

### Visualization of Neuronal Recording and Injection Sites using Quantitative Susceptibility Mapping (QSM).

To localize our recording and injection sites within the SNr, which is challenging to visualize with conventional T1- and T2-weighted MRI (MRI) due to its signal similarity to surrounding structures, we used QSM, an advanced MRI technique that exploits the paramagnetic properties of iron-rich regions (*SI Appendix*, Fig. S3*A*). As shown in prior work (42), QSM provides clear visualization of subcortical structures, including the iron-rich SNr, in macaques. Our QSM pipeline included multiecho gradient echo acquisition, phase unwrapping, background field removal, and dipole field inversion to create susceptibility maps.

QSM offered superior visualization of basal ganglia structures compared to T1- and T2-weighted imaging (*SI Appendix*, Fig. S3*B*). High susceptibility values were observed in the lateral SNr, corresponding to the substantia nigra pars lateralis (SNpl), which showed the strongest QSM signal and the highest concentration of task-related neurons. This region also served as the site for targeted pharmacological manipulations. The enhanced contrast in QSM images allowed precise confirmation of recording and injection sites, ensuring the anatomical specificity of our experimental interventions.

## Discussion

We examined the role of the SNr in action selection and suppression, focusing on glutamatergic inputs and the differentiation between reactive and proactive inhibition. We addressed two major questions: 1) whether individual SNr neurons in primates bidirectionally modulate activity to facilitate and suppress actions, and 2) whether glutamatergic input provides the causal drive for these inhibitory processes, and if so, whether it predominantly supports reactive or proactive inhibition. Using electrophysiological recordings, behavioral tasks, and pharmacological manipulations, we provided evidence relevant to these questions. Recordings from SNr neurons in macaque monkeys during a sequential choice task revealed bidirectional coding: Neurons decreased firing rates for desirable targets and increased firing for undesirable ones. This dynamic modulation was consistent across contexts and linked to monkeys’ choices, highlighting the SNr’s potential role in both facilitating and suppressing actions. Pharmacological blockade of glutamatergic inputs to the lateral SNr disrupted saccadic control, altering reaction times and rejection behaviors. These findings support a contributory role for excitatory inputs in behavioral inhibition and help close an important gap in understanding SNr function.

### Does the Lateral SNr Mediate Reactive, Proactive, or Both Forms of Inhibition?

Behavioral inhibition is classified into proactive and reactive forms (41). Proactive inhibition involves anticipatory action suppression to achieve a goal, enabling planned restraint. Reactive inhibition is the immediate suppression of actions in response to unexpected stimuli, allowing rapid adjustment to environmental changes. In our sequential choice task, rejecting a bad object represents proactive inhibition, as it serves the goal of accepting a good object for a reward. However, our findings indicate that the lateral SNr primarily supports reactive, not proactive, inhibition.

Several lines of evidence support this conclusion. First, glutamate receptor antagonist injections into the lateral SNr significantly shortened saccadic reaction times to both contralateral good and bad objects in the choice task (Fig. 4*A*). If the lateral SNr were solely responsible for proactive inhibition, antagonist injections would likely impair bad object rejection, leading to more saccades toward or acceptance of bad objects. Instead, the shortened reaction times for both good and bad objects suggest a disruption of reactive inhibition, which normally suppresses reflexive saccades to any suddenly appearing stimulus, regardless of its value. Blocking glutamatergic input to the SNr likely weakened this initial reactive suppression, resulting in faster but less controlled saccades.

Second, the similar patterns of SNr activity observed during “return” and “stay” rejections further support the role of the lateral SNr in reactive inhibition. Both “return” (making a saccade toward the bad object and then quickly back to the center) and “stay” (maintaining central fixation) represent successful rejection of the bad object. However, they differ significantly in their motor output: “Return” involves a saccade, while “stay” requires its suppression. The fact that SNr activity increased similarly during both types of rejection (Fig. 3) indicates that this activity is not simply related to the execution or suppression of a specific movement (i.e., a saccade). Instead, it likely reflects a more general process of reactive inhibition, triggered by the appearance of the unexpected (and, in this context, undesirable) stimulus.

Third, the timing of lateral SNr activity is more consistent with reactive inhibition. During bad object rejections, peak SNr activity precedes return saccade onset (*SI Appendix*, Fig. S2), suggesting a role in the immediate suppression of a reflexive saccade, rather than the execution of a planned, proactive rejection.

We propose that reactive and proactive inhibition both operate in the choice task but serve distinct roles. Reactive inhibition initially suppresses reflexive saccades to all presented objects, providing a brief window for object evaluation before a response is made. If the object is deemed bad, proactive inhibition engages to reject it.

This hypothesis is supported by saccadic reaction times (Fig. 1*D*). Across all monkeys, saccades to good objects were faster than to bad objects, indicating that object evaluation occurs *before* saccade initiation. If evaluation followed reflexive saccades, no reaction time difference would be expected.

### SNr Input and Reactive Inhibition.

Hikosaka and Wurtz (1984) demonstrated that SNr neurons tonically inhibit SC neurons via GABAergic projections, with pauses in SNr activity disinhibiting SC neurons for saccade generation (41). While establishing the SNr’s role as a key output structure, their work focused on the output pathway. Our study builds on this by examining glutamatergic inputs to the lateral SNr, likely from the subthalamic nucleus (STN) via the hyperdirect pathway. Our pharmacological results support the hypothesis that these inputs are crucial for reactive inhibition—the rapid, stimulus-driven suppression of prepotent responses, as evidenced by increased fixation break errors after glutamate receptor blockade. Thus, in contrast to Hikosaka and Wurtz’s focus on general saccade control, our study highlights the importance of SNr inputs in reactive inhibition. While the cortico-STN-SNr pathway likely mediates this, the relative contributions of the hyperdirect and indirect pathways remain to be determined. Our findings expand the SNr’s role beyond a simple output relay, positioning it as a critical node integrating excitatory inputs to rapidly suppress unwanted actions.

### Potential Sources of Excitatory Input to the Lateral SNr.

Identifying the source of excitatory inputs to the lateral SNr that mediate behavioral inhibition is essential for interpreting our findings. Our results provide direct evidence of the causal role of glutamatergic transmission in mediating behavioral inhibition: Blocking glutamatergic transmission shortened saccadic reaction times in the choice task and increased fixation break errors in the fixation task.

While these results underscore the importance of glutamatergic inputs to the lateral SNr, the precise source of these inputs has yet to be definitively identified. The subthalamic nucleus (STN) emerges as the most likely candidate, based on robust anatomical evidence supporting a direct, glutamatergic STN-SNr projection. This pathway is a critical component of both the indirect and hyperdirect basal ganglia circuits in primates (43–48). Additionally, physiological studies have shown that STN neurons project to and can excite SNr neurons (49, 50). Optogenetic studies in rodents further support the involvement of STN excitatory projections in behavioral inhibition (38, 51).

Moreover, Matsumura et al. characterized multiple types of task-related activity in STN neurons, including those associated with eye fixation, saccades, visual responses, and target- and reward-related processing (52). Building on these findings and our current results, we hypothesize that the glutamatergic input to the SNr that drives increased activity during reactive inhibition likely originates from a subset of STN neurons, potentially including those involved in visual responses and fixation-related activity.

However, contributions from other sources cannot be definitively excluded. While the cortico-STN-SNr hyperdirect pathway is well established, some studies in rodents and primates have reported sparse, direct cortical projections to the SNr (53, 54, 55). Nevertheless, these projections appear to be less dense and functionally less significant than the STN-SNr pathway.

Although our current findings strongly implicate the hyperdirect pathway in mediating the reactive suppression of reflexive saccades in the lateral SNr, we cannot rule out contributions from the indirect pathway and proactive inhibition. Additionally, proactive inhibition may be primarily mediated by other SNr subregions, as our study focused predominantly on the lateral SNr, where saccade-related neurons have been traditionally reported. We did not extensively sample from the medial SNr or other subregions.

Future studies employing pathway-specific manipulations in primates, ideally integrating optogenetic stimulation of the STN with concurrent recordings in the lateral SNr, are necessary to confirm the STN as the primary source of the glutamatergic input mediating behavioral inhibition. Such studies could also delineate the relative contributions of the STN, motor cortex, and other potential sources to the excitatory drive of the lateral SNr in primates.

### Comparative Analysis of Lateral SNr Function: Conserved Bidirectional Coding?

Our study revealed that most lateral SNr neurons (91.5%, 118/129) exhibit bidirectional coding, modulating activity to encode both saccade facilitation and suppression. This aligns with previous findings in other species. For instance, Schmidt et al. observed that 55.6% (10/18) of rat lateral SNr neurons decreased activity during planned actions and increased activity during successful cancellations in a stop-signal task (33, 56). This suggests that bidirectional modulation related to action control may be evolutionarily conserved.

In contrast, Jiang et al., studying anesthetized cats, reported distinct SNr neuron populations projecting to the ipsilateral and contralateral SC, with inhibitory and excitatory responses to contralateral visual stimuli, respectively (57). This suggests functional segregation of SNr neurons rather than bidirectional coding within individual neurons. However, anesthesia profoundly alters neuronal activity and synaptic transmission, potentially masking true functional properties. Differences in experimental conditions (anesthetized cats vs. awake rats and monkeys in our study) likely account for these discrepancies.

These contrasting results emphasize the need to consider species differences and experimental conditions when studying SNr function. The disparity between findings in awake and anesthetized animals highlights how consciousness can profoundly influence neural circuit dynamics. Further research in awake preparations across multiple species is essential to determine whether bidirectional coding in the lateral SNr is a conserved feature and to clarify the effects of anesthesia on SNr responses. This will enhance our understanding of the principles governing SNr function in action control. The evolutionary origins and conservation of bidirectional coding in the SNr remain open questions. Investigations using advanced techniques like optogenetics or high-resolution imaging in primate models are crucial to unravel these mechanisms.

### Limitations and Future Directions.

While our findings provide important insights into the role of the SNr and its glutamatergic inputs in reactive inhibition, several limitations should be acknowledged when interpreting the results and formulating strong conclusions about specific neural circuits.

First, although our electrophysiological recordings reveal strong correlations between SNr neuronal activity and behavioral events (e.g., during action selection and suppression), these data are inherently correlational. Establishing definitive causality between specific SNr firing patterns (e.g., increased firing) and the suppression of specific actions requires caution, as this activity could reflect other related processes not directly measured or manipulated in our electrophysiological experiments.

Second, our pharmacological manipulation, while providing causal evidence for the necessity of glutamatergic transmission in the lateral SNr for reactive inhibition, lacks pathway specificity. As discussed previously (“Potential sources of excitatory input to the lateral SNr”), the STN is the most likely source based on converging evidence. However, this manipulation cannot experimentally distinguish STN inputs from other potential glutamatergic sources, such as sparse direct cortical projections (53–55). Thus, our conclusion regarding the STN’s primary role relies heavily on integrating our findings with the existing literature.

Third, contributions from different basal ganglia pathways (hyperdirect vs. indirect) likely converge on the SNr. As detailed in the preceding section, both anatomical and functional evidence implicate the hyperdirect pathway, particularly given its role in rapid motor suppression. Nonetheless, our current methods cannot definitively parse the relative contributions of the hyperdirect versus indirect pathways to the observed SNr activity or the effects of glutamate blockade, and contributions from the indirect pathway cannot be excluded, especially concerning proactive aspects of inhibition.

Fourth, our recordings were concentrated in the lateral SNr. As noted earlier, other SNr subregions might mediate different functions (e.g., proactive inhibition) or receive different input compositions. Therefore, generalizing our findings regarding the dominance of reactive inhibition and the role of glutamatergic inputs to the entire SNr function requires further investigation across different subregions and behavioral contexts.

Addressing these limitations definitively would require future studies in primates employing pathway-specific investigation techniques. For instance, optogenetic stimulation or DREADDs (Designer Receptors Exclusively Activated by Designer Drugs) specifically targeting STN-SNr projections, cortico-SNr projections, or components of the indirect pathway, combined with concurrent SNr recordings and behavioral assessments, would be necessary to precisely determine the origin and contribution of different excitatory inputs to SNr function during behavioral control. These approaches represent the causal experiments that will be required to confirm pathway-specific mechanisms.

## Materials and Methods

The behavioral tasks and experimental procedures were identical to those in our previous investigation of striatal activity during choice and fixation tasks (40). Below, we briefly outline essential procedures and refer readers to our earlier work for additional details.

### Animal Preparation.

All procedures were approved by the National Eye Institute Animal Care and Use Committee and complied with the Public Health Service Policy on Laboratory Animal Care. Three male macaque monkeys (Macaca mulatta, 8 to 10 kg), referred to as Monkeys Ch, Cr, and Sp, were used. Monkeys Cr and Sp also participated in our previous study (40). Under isoflurane anesthesia and sterile conditions, we implanted a plastic head holder and recording chambers. After recovery, monkeys were trained on oculomotor tasks. During experimental sessions, their heads were fixed, and eye movements were tracked using an infrared device (EyeLink 1000, SR Research) at 1,000 Hz. Water intake was regulated to maintain task motivation. For detailed surgical and postoperative procedures, see our previous report (40).

### Behavioral Results.

Experiments were conducted in a dark, soundproof room. Visual stimuli were presented on a screen using an LCD projector (PJ658, ViewSonic). Task control and data acquisition were managed using our custom-written visual C++ based software. As in our previous study (39), we used two behavioral tasks: a choice task and a fixation task.

### Choice Task.

The structure and timing of the choice task matched our previous study. Briefly, one of six scene-object sets was randomly selected for each recording session (*SI Appendix*, Fig. S1). Each set included four scenes with two fractal objects per scene: one “good” (rewarded) and one “bad” (nonrewarded). In scenes 1 and 2, object values were stable, while in scenes 3 and 4, values were reversed, dissociating visual features from value coding.

On each trial, after scene presentation (1,000 ms) and central fixation (700 ms), either a good or bad object appeared at one of six peripheral locations (15° eccentricity). Monkeys could accept the object by making a saccade and maintaining fixation (>400 ms) or reject it through three behaviors: returning to center after a brief saccade (“return”), maintaining central fixation (“stay”), or looking away (“other”). Accepting good objects earned juice rewards (0.4 mL). After rejection, another object was presented until acceptance.

### Fixation Task.

After isolating task-related neurons in the choice task, we conducted a fixation task to examine SNr activity in saccade suppression. Good and bad objects from scene 1 of the choice task were presented sequentially (2 to 4 times, 400 ms duration, 400 ms interval) while monkeys maintained central fixation. Successful fixation through the sequence earned a reward, while saccades toward objects were counted as fixation break errors.

### MRI.

After implanting recording chambers, an MRI was performed to map cerebral structures and grid apertures. A gadolinium-enhanced contrast medium (Magnevist, Bayer Healthcare Pharmaceuticals) was introduced into the chambers. Recording sites were identified using a high-definition 3 T MR system (MAGNETOM Prisma; Siemens Healthcare) with three-dimensional T1-weighted (T1w, MPRAGE) and T2-weighted (T2w, SPACE) sequences (0.5 mm voxel size).

Because conventional T1w and T2w images provided poor contrast between the SNr and surrounding structures, QSM was used to enhance SNr visualization (42, 58, 59). QSM images were reconstructed from phase images acquired with a 3D multiecho gradient echo sequence (repetition time: 50 ms; echo times: 3.7, 10.1, 16.7, 23.4, 30.0, 36.6, 43.2 ms) (40). The QSM reconstruction pipeline included multiple steps:

1. Phase Unwrapping: Phase images were unwrapped.
2. Background Field Removal: High-pass filtering was applied to the phase images.
3. Dipole Inversion: Dipole field inversion was performed to generate quantitative susceptibility maps.

These steps were implemented using the morphology-enabled dipole inversion toolbox (http://pre.weill.cornell.edu/mri/pages/qsm.html) (60) in MATLAB 2019 (The MathWorks, Inc., Natick, MA).

### Neuronal Recording Procedure.

Single-unit activity was recorded from the SNr using tungsten electrodes (1 to 9 MΩ; Frederick Haer & Co.; Alpha Omega Engineering). Electrodes were advanced through stainless steel guide tubes with a hydraulic micromanipulator (MO-973A, Narishige). Neural signals were amplified, bandpass filtered (0.3 to 10 kHz; A-M Systems), and digitized at 40 kHz. Individual neurons were isolated online using custom voltage–time window discrimination software (Blip). Recording began once stable isolation was achieved, and the neuron showed activity modulation following target onset in the choice task.

### The Glutamatergic Antagonist Injection Procedure.

To inhibit excitatory projections to the SNr, we injected a mixture of CPP (C104, Sigma-Aldrich) and NBQX (N183, Sigma-Aldrich) into the lateral SNr, where task-related neurons were concentrated. The high baseline firing rate of SNr neurons allowed the precise localization of the dorsal SNr edge with the electrode tip. Prior to injection, neuronal activity was recorded using a custom Injectrode (61).

Monkeys first performed the choice and fixation tasks to collect preinjection control data. Then, 1 μL of a 5 to 10 mM mixture of CPP and NBQX was injected at 0.2 μL/min using a remotely controlled infusion pump (PHD ULTRA, Harvard Apparatus). Concentrations were based on a previous study (62), with higher levels used to assess the effects of excitatory projections on behavior. After injection, monkeys repeated the choice and fixation tasks to evaluate the effects of CPP and NBQX (5 to 90 min postinjection). As a control, 1 μL of saline was injected into the same SNr sites.

For statistical analyses, data from 60 to 90 min postinjection of CPP, NBQX, and saline were used. However, for one data point from monkey Sp (10 mM CPP and NBQX), analysis was performed at 20 min postinjection because the effects were too strong for task continuation.

### Data Analysis and Statistical Analysis.

All behavioral and neurophysiological data were preprocessed using MATLAB 2022b (MathWorks, Natick, MA). The sample size was not predetermined statistically but guided by previous studies on recorded SNr neurons (28).

### Behavior Data Analysis.

In the choice task, saccade onsets were defined as eye velocity exceeding 40°/s within 400 ms of target onset. Reaction times for good and bad objects (Fig. 1*D*) included only initial saccades toward objects, excluding return movements. Welch’s *t* test compared reaction times across conditions. Fisher’s exact test was used to compare “stay” responses between stable (scenes 1 and 2) and flexible (scenes 3 and 4) value conditions (Fig. 1*E*). In the fixation task, saccades toward presented objects were classified as fixation break errors.

### Neuronal Data Processing.

Neuronal data were aligned with event initiation (scene, target, and saccade onset). Peristimulus time histograms (PSTHs) were calculated in 1-ms bins and smoothed with a Gaussian filter (σ = 20 ms). Neuronal activity was Z-transformed by subtracting the baseline firing rate (average firing during the 500 ms before event onset) from the smoothed PSTH and dividing it by the PSTH’s SD (63, 64). The time course of responses was analyzed for each condition using these Z-transformed PSTHs.

To quantify neurons showing significant modulation to good and/or bad objects (Fig. 2*G*), neural activity was compared between baseline and poststimulus periods for contralateral targets in scene 1. The baseline period was 200 ms before the target onset, and the poststimulus period was 200 ms starting 100 ms after the target onset. For each neuron, two-sample *t* test (α = 0.05) compared normalized firing rates between these periods for good and bad objects. Neurons were classified as responding to 1) good objects only, 2) bad objects only, or 3) both. These classifications were visualized using a Venn diagram (Fig. 2*G*).

### Statistical Modeling.

To compare normalized neuronal activity and behavioral parameters (e.g., saccade reaction times) before and after injections, we used linear mixed-effects models (LMMs) and generalized linear mixed-effects models (GLMMs). These hierarchical models incorporate random effects for individual subjects and neurons, accounting for repeated measurements. By modeling fixed and random effects, LMMs/GLMMs reduce type I errors and better represent the data structure (65).

LMMs were used to analyze Z-transformed SNr neuronal activity under various conditions (Figs. 2 and 3 and *SI Appendix*, Fig. S2). GLMMs compared saccade reaction times, proportions of chosen actions for bad objects in the choice task, and fixation break errors in the fixation task (Fig. 4) before and after injection. For all LMM and GLMM tests, we compared full models with explanatory variables as fixed effects and random effects for monkey, neuron, or session IDs (null model). Detailed model specifications are provided in Supplementary Methods.

We used a parametric bootstrap method to assess model goodness of fit, performing 10,000 iterations and computing the p-value from the deviance difference between models. If the full model demonstrated significant fit, post hoc pairwise *t* test with Bonferroni correction were conducted to explore differences. For LMM and GLMM analyses, we used the lme4 (66), pbkrtest (67), emmeans (68), and brms (69) packages in RStudio.

## Supplementary Material

Appendix 01 (PDF)

## Data Availability

All data used for the statistical tests presented in this study have been deposited in the Zenodo database and are publicly available (70).
